# Novel biotechnological glucosylation of high-impact aroma chemicals, 3(2H)- and 2(5H)-furanones

**DOI:** 10.1038/s41598-019-47514-9

**Published:** 2019-07-29

**Authors:** Isabelle Effenberger, Thomas Hoffmann, Rafal Jonczyk, Wilfried Schwab

**Affiliations:** Biotechnology of Natural Products, Technische Universität München, Liesel-Beckmann-Str. 1, 85354 Freising, Germany

**Keywords:** Bioinspired materials, Expression systems

## Abstract

Glucosyltransferases are versatile biocatalysts to chemically modify small molecules and thus enhance their water solubility and structural stability. Although the genomes of all organisms harbor a multitude of glucosyltransferase genes, their functional characterization is hampered by the lack of high-throughput *in-vivo* systems to rapidly test the versatility of the encoded proteins. We have developed and applied a high-throughput whole cell biotransformation system to screen a plant glucosyltransferase library. As proof of principle, we identified 25, 24, 15, and 18 biocatalysts transferring D-glucose to sotolone, maple furanone, furaneol and homofuraneol, four highly appreciated flavor compounds, respectively. Although these 3(2H)- and 2(5H)-furanones have extremely low odor thresholds their glucosides were odorless. Upscaling of the biotechnological process yielded titers of 5.3 and 7.2 g/L for the new to nature β-D-glucopyranosides of sotolone and maple furanone, respectively. Consequently, plant glucosyltransferase show stunning catalytic activities, which enable the economical production of novel and unexplored chemicals with exciting new functionalities by whole-cell biotransformation.

## Introduction

**Figure 1:**
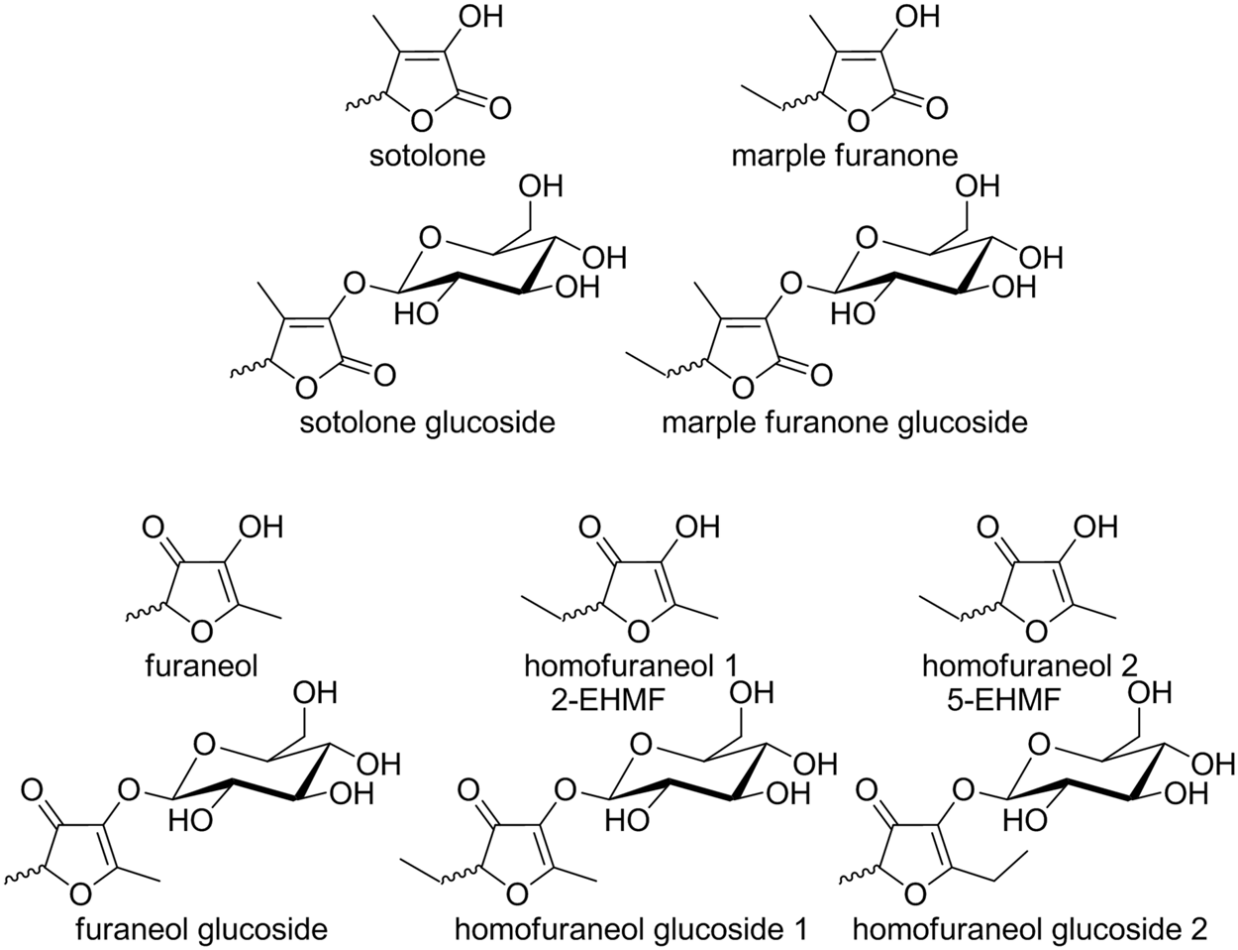
Chemical structures of substrates and glucosides.

Sotolone (4,5-dimethyl-3-hydroxy-2(5H)-furanone) is a naturally occurring chiral lactone of industrial significance and is considered a high impact aroma chemical (Fig. 1)^1^. The 2(5H)-furanone shows characteristic organoleptic properties with the typical smell of curry and fenugreek at high concentration and caramel, or burnt sugar at lower concentration and has an extremely low odor threshold of 0.8 and 89 ppb for the (+)-(S)- and (−)-(R)-enantiomer, respectively^2–4^. Sotolone was first described as a breakdown product of the amino acid threonine^5^ and was later proposed as the flavor principle of seasonings prepared from plant protein hydrolysates^6^. It has since been identified in many foods, including coffee, aged sake and rum, flor sherry, and wines^7–12^. Besides, sotolone is the key aroma component of fenugreek and lovage^13^ and has been found in the dried fruiting bodies of the mushroom *Lactarius helvus*^14^. The remarkable contribution of sotolone to the aroma of different foods explains the numerous studies that focused on identifying the generation pathways of this exceptional aroma chemical^15,16^. Both, enzymatic and chemical reactions are responsible for the formation^17,18^.

The ethyl analogue of sotolone, 5-ethyl-3-hydroxy-4-methyl-2(5H)-furanone (maple furanone, adhexon; Fig. 1) is one of the key contributors to the aroma of roasted coffee and occurs naturally in fruits such as blackberry, raspberry and blueberry and was found in wines and beers^19–21^. Maple furanone was initially synthesized in 1957 and later identified in hydrolyzed soy protein in 1980, and has been named after its powerful maple-caramel aroma and taste, which is reminiscent of maple syrup^22^. The odor thresholds were assessed at 0.02−0.04 ng/L in air and 0.1 μg/L in water. Thus, it is one of the most outstanding modern flavor materials. Maple furanone is a key organoleptic note in soy sauce and one of the most powerful flavor chemicals known to man. It is thought to arise from the reaction between α-ketobutyric acid and propanal^20^.

Aroma glycosides are a class of compounds, which have recently attracted much attention from the industry as they can function as proflavor and profragrances in a number of food and cosmetics applications, respectively^23–25^. They constitute novel and important delivery systems for the controlled release of volatile compounds. Aroma glycosides consist of an aroma component (aglycone) linked to a carbohydrate moiety (glycone) and are formed by uridine diphosphate-sugar dependent glycosyltransferases in cells (UGTs)^26^. Aroma glycosides are abundant in nature and have been isolated from plants (flowers, leaves, roots and fruits), insects and microorganism^27–34^.

The chemical synthesis of glycosides is well established, but is still a labor-intensive task, which involves multiple protection and deprotection steps to reduce the formation of unwanted side products. In all organisms, however, UGTs selectively catalyse the transfer of a sugar from activated nucleotide-sugar donor molecules (usually UDP glucose in plants) to an acceptor molecule without the need for protective groups. Biocatalytic processes involving UGTs thus represent an interesting alternative to chemical synthesis of glycosides^24,25,35,36^.

Sugar conjugation results in increased stability and water solubility as consequence of enhanced polarity and has also a major impact on biological activity and toxicity^37^. Because of their sessile life style, plants have evolved different mechanisms to cope with environmental threats involving a large gene family of UGTs for detoxification of endogenous and exogenous substances^38,39^. Consequently, plants are a particular rich source of unexplored UGT genes.

Recently, the first furaneol/homofuraneol glucosyltransferases from grapevine and strawberry were reported^40–42^. Due to the structural similarity of the furaneols with sotolone, and maple furanone (Fig. 1) we assumed that UGTs acting on 3(2H)-furanones might also be able to glucosylate 2(5H)-furanones. Thus, the aim of this study was to develop a rapid screening system for the testing of plant UGTs *in vivo*. As proof-of-principle, we selected the four highly appreciable flavor compounds as substrates to identify UGTs acting on furanones.

Here, we describe the systematic search for plant UGTs of different origins that are able to convert intensely smelling aroma compounds into nonvolatile proflavors. The identification of numerous biocatalysts enabled the establishment of the first biotechnological process for the production of furanone ß-D-glucosides.

## Results and Discussion

### Identification of 3(2H)- and 2(5H)-furanone:UDP-glucose glucosyltransferases

**Figure 2:**
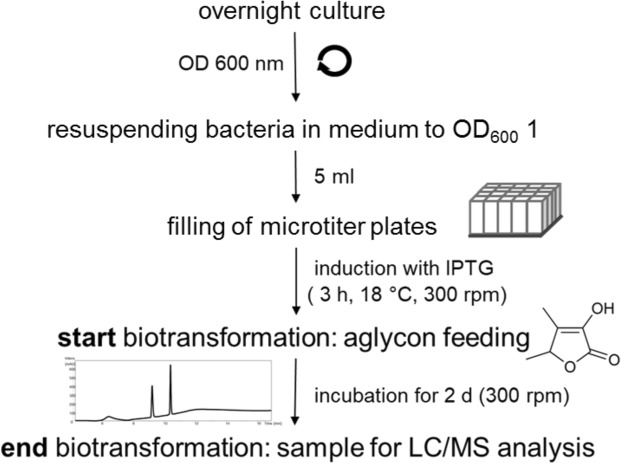
Workflow of the high-throughput screen to identify glucoside producing UGTs by biotransformation.

**Figure 3:**
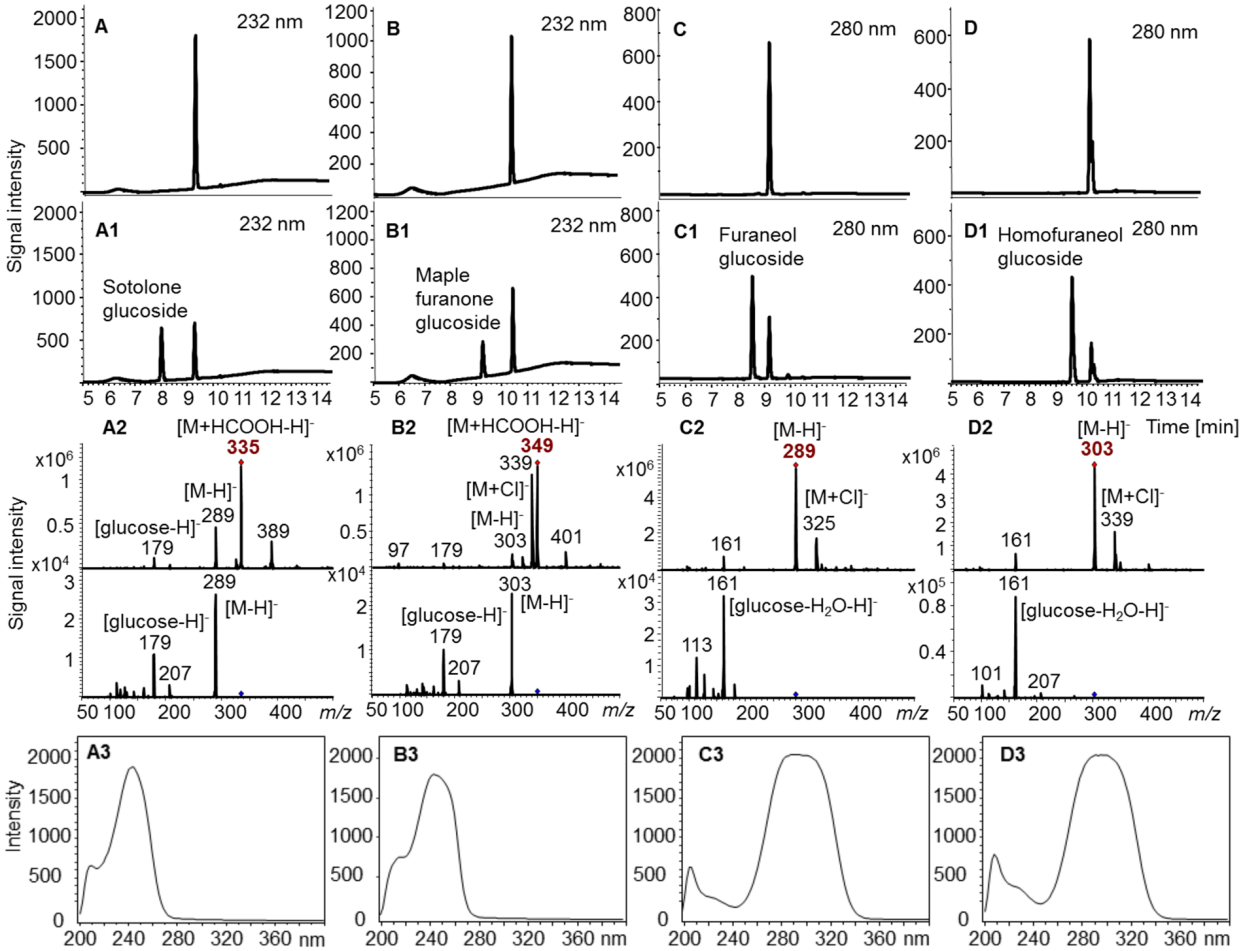
Production of glucosides by biotransformation using UGT-transformed *E*. *coli* W cells. Sotolone (**A**), maple furanone (**B**), furaneol (**C**) and homofuraneol (**D**) were used as substrates. Glucoside formation was monitored by LC-UV-MS (negative mode). UV-chromatograms of substrates and products after biotransformation using *E*. *coli* W wild-type (**A**–**D**), UGT84A45- (**A1**), UGT71K3 transformed cells (**B1**), and UGT72B27-transformed cells (**C1**,**D1**). Mass spectra, product ion spectra and UV spectra of sotolone glucoside (**A2**,**A3**), maple furanone glucoside (**B2**,**B3**), furaneol glucoside (**C2**,**C3**), and homofuraneol glucoside (**D2**,**D3**).

Since furanones are potent flavoring agents, we envisioned the development of a biocatalytic process for the production of furanone glucosides based on *E*. *coli* whole-cell biocatalysts expressing recombinant plant UGTs. To find efficient enzymes a UGT library consisting of 49 plant enzymes including catalytically active proteins from grapevine (*Vitis vinifera*), strawberry (*Fragaria* spp), raspberry (*Rubus idaeus*), tobacco (*Nicotiana benthamina*), and Arabidopsis (*Arabidopsis thaliana*), were screened in 24-well plate format (Fig. 2). Due to the fast growth of *E*. *coli* and high specific oxygen consumption rates, the oxygen-transfer rate is a major criterium when choosing the type of microplate, culture volume, and shaking conditions. The use of a 5-ml bacterial solution in 25-ml wells allowed the cultivation of *E*. *coli* without affecting the growth of the bacteria by oxygen deprivation. Expression of recombinant enzymes in the *E*. *coli* cells harboring the *UGT* genes was induced with isopropyl ß-D-1-thiogalactopyranoside (IPTG) and furanone substrates were added to initiate the biotransformation experiment. Glucoside products were identified in the supernatant by LC-UV-MS analysis (Fig. 3; Supplementary Fig. S1).

**Figure 4:**
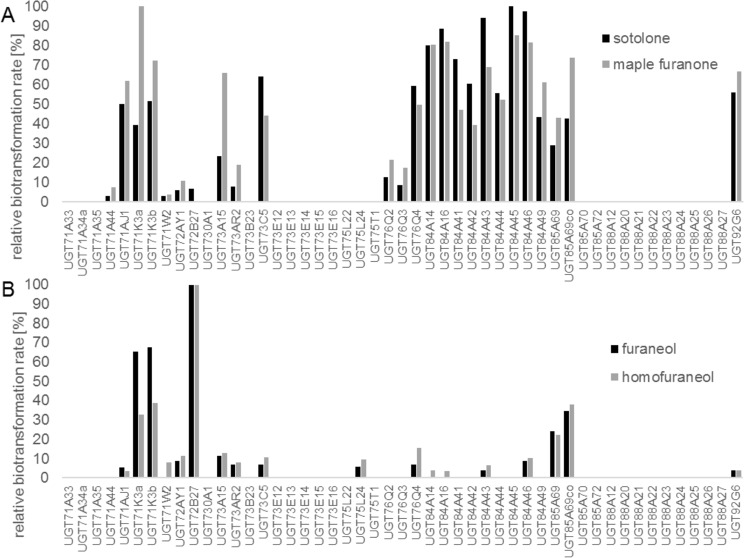
Relative biotransformation rate of UGTs in % towards sotolone (**A**, black bars), maple furanone (**A**, grey bars), furaneol (**B**, black bars) and homofuraneol (**B**, grey bars) using a UGT library transformed in *E*. *coli* W cells. Glucoside formation was monitored by LC-UV-MS (negative mode). Peak areas at 232 nm (sotolone and maple furanone glucoside) and 280 nm (furaneol and homofuraneol glucoside) were used and the UGT with the highest value was set to 100%; co, codon-optimized.

Out of 49 different plant UGTs, used as *E*. *coli* whole-cell biocatalyst, 25 and 24 transformed sotolone and maple furanone, respectively (Fig. 4) while furaneol and homofuraneol were converted by 15 and 18 UGTs, respectively. UGTs of families 71, 72, 73, 76, 84, 85, and 92 glucosylated the 2(5H)-furanones and enzymes of families 71, 72, 73, 75, 76, 84, 85, and 92 transferred D-glucose onto 3(2H)-furanones. While a number of UGTs transformed sotolone and maple furanone equally efficiently, especially those of family 84, one enzyme UGT72B27 stood out for furaneol and homofuraneol glucosylation (Fig. 4). With few exceptions, the structural homologues sotolone and maple furanone as well as furaneol and homofuraneol showed similar reactivities towards the individual biocatalysts.

Glycosyltransferases of the UGT84A clade are known to produce glucose esters from benzoic and cinnamic acid derivatives and are involved, among others in the production of galloylated plant metabolites^43,44^. Therefore, it was unexpected that members of this class efficiently formed glucosides of sotolone and maple furanone. UGT71K3a and UGT71K3b catalyzed the glucosylation of diverse hydroxycoumarins, naphthols, flavonoids, phloroglucinols and pelargonidin^45^ as well as furaneol in strawberry fruit^41^. UGT72B72, which was a superior biocatalyst for the production of furaneol and homofuraneol in this study, glucosylated the natural stilbene trans-resveratrol and the smoke-derived volatiles guaiacol, syringol, methylsyringol, and methylguaiacol in *Vitis vinifera*^46^.

### Characterization of furanone glucosides

The pseudo-molecular ions of the transfer products from sotolone were observed at *m/z* 335 [M + HCOOH-H]^−^ and 289 [M-H]^−^ (Fig. 3) while products formed from maple furanone showed pseudo-molecular ions at *m/z* 349 [M + HCOOH-H]^−^, 339 [M + Cl]^−^, and 303 [M-H]^−^. Similarly, [M-H]^−^ and [M + Cl]^−^ ions were observed at *m/z* 289 and 325 for the transfer product from furaneol, respectively and *m/z* 303 and 339 for homofuraneol products, respectively. Remarkably, the product ion spectra (MS2) of the furanone glucoside products showed a neutral loss of the aglycone resulting in [glucose-H]^−^ and [glucose-H_2_O-H]^−^ ions for the 2(5H)- and 3(2H)-furanone glucosides, respectively. Generally, MS2 spectra of glucosides are characterized by a neutral loss of the glucose moiety^47^.

The structures of the transglucosylated products were proposed considering the racemic forms of the substrates and the tautomerism of homofuraneol (Fig. 1) and their details analyzed by NMR spectroscopy (Supplementary Tables S1–S3). For analyses of the products by NMR, glucosides were produced on larger scale in 2 L shaking flasks and purified by solid phase extraction. ^1^H- and ^13^C-NMR signals of the products were assigned by studies of ^1^H-^1^H COSY, HMQC, and HMBC spectra. The ^1^H-NMR spectra of the glucosylated furanones showed the characteristic signals due to β-D-glucopyranosyl residues and the ^13^C-NMR spectra exhibited signals for the anomeric carbons from 100.79 ppm (sotolone glucoside, C1’) to 105.63 ppm (homofuraneol glucoside, C1”). The signals for the anomeric carbons of the furanone glucosides were split into two signals. Since all furanones were racemates^22^, the signals were considered to reflect the production of two diastereomers by conjunction with β-D-glucopyranose. The similar ^13^C signal intensities for the anomeric carbons showed that the substrate enantiomers were only slightly discriminated. Splitted signals were also observed for almost all carbons. Additionally, there were two sets of signals for homofuraneol glucoside, which derived from two tautomeric isoforms, 2-ethyl-4-hydroxy-5-methyl-3(2H)-furanone (2-EHMF) and 5-ethyl-4-hydroxy-2-methyl-3(2H)-furanone glucoside (5-EHMF; Supplementary Tables S3). The ^1^H-NMR signals of the homofuraneol moieties were separately detected, and their correlation with the carbon signals were determined in the HMQC spectrum. However, the signals of the glucosyl residues strongly overlapped. Based on the NMR data, the products formed by the UGT whole-cell biocatalysts were established to be furanone β-D-glucopyranosides.

Although numerous attempts have been undertaken to identify sotolone glucoside by LC-MS analysis in extracts of plants known to produce the aglycone, all investigations have failed (not shown). Up to date, sotolone and maple furanone glucoside can be considered as new-to-nature products while furaneol glucoside has been isolated from a variety of fruits including strawberry and grapes^41^. The tautomeric homofuraneol α-D-glucopyranosides have been synthesized by sucrose phosphorylase^48^ but the β-D-glucopyranosides have not been found in nature until to date.

### Biocatalytic large-scale production of sotolone β-D-glucopyranoside

**Figure 5:**
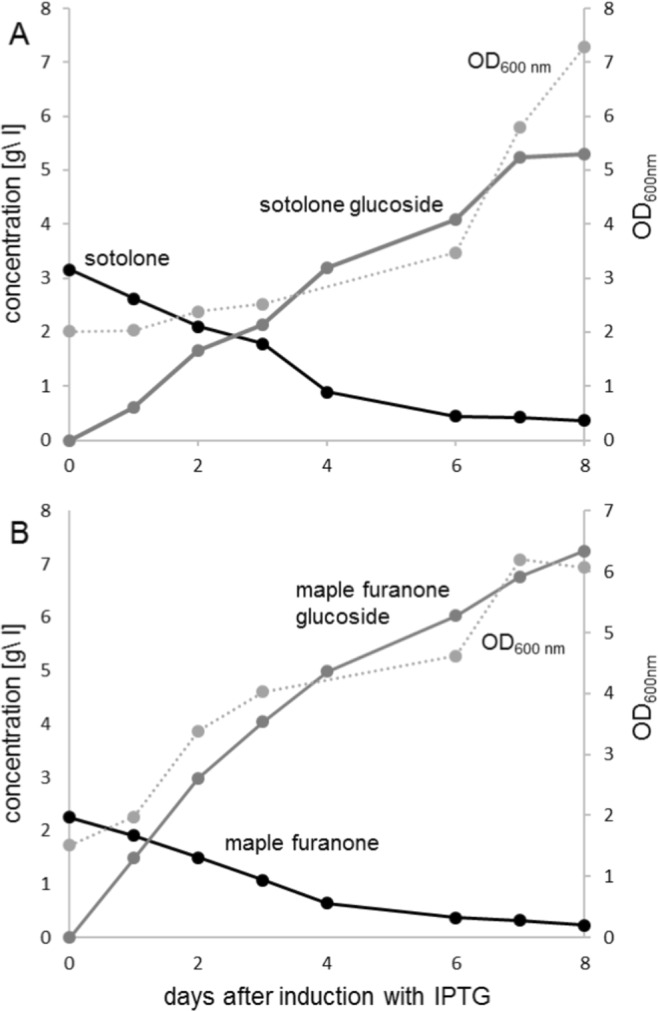
Batch fermentation for the production of sotolone glucoside (**A**) and maple furanone glucoside (**B**). UGT84A45 and UGT71K3a were used for the production of sotolone glucoside and maple furanone glucoside, respectively. Mean values of two replicates are shown.

To explore the potential of *E*. *coli* for large-scale production of sotolone and maple furanone glucosides, UGT-expressing bacterial cells were used as whole-cell biocatalysts in 2-L-shaking flasks. UGT84A45 and UGT71K3a were chosen for the production of sotolone and maple furanone, respectively. The screening experiments (Fig. 4) had shown maximum activity of the selected enzymes for the target molecules. More and more furanones were glucosylated in the course of the reaction. The yields of β-D-glucopyranosides of sotolone and maple furanone in the supernatant at 8 days after IPTG induction were 5.3 and 7.2 g/L, respectively (Fig. 5). The glucosides produced by the whole-cell biocatalysts could be readily purified by solid phase extraction from the supernatant of the cultures. The furanone glucosides were completely recovered by methanol elution of the resin and colored impurities were removed by activated carbon as described for the purification of sugars^49^.

A comprehensive overview of whole-cell systems for UDP-sugar based glycosylation focusing on small molecules has been shown that UGTs have only been successfully applied for the production of oligosaccharides in terms of titers (up to 188 g/L). To date, glycosides derived from low-molecular weight metabolites were produced about three orders of magnitude lower^50^. Thus, whole-cell biotransformation of furanones by *E*. *coli* cells harboring plant UGTs offer an interesting alternative for chemicals synthesis. One probable explanation of the relatively high titers of the furanone glucosides is the high water solubility and low toxicity of the substrates, which do not hinder the efficient production of the corresponding glucosides. As further improvements are possible, an economic bioprocess is within reach.

### Enzymatic hydrolysis

**Figure 6:**
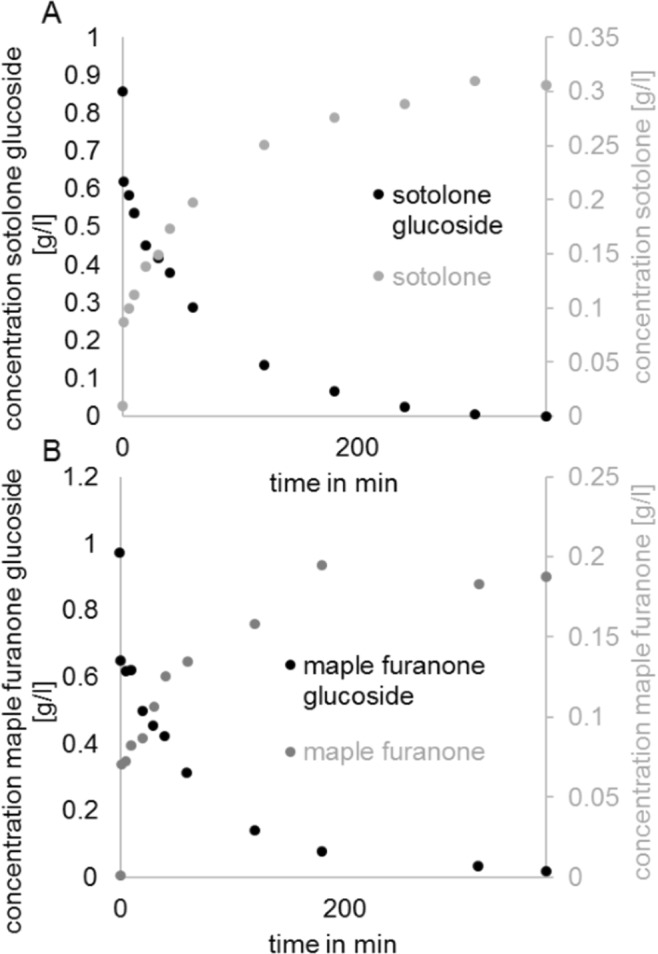
Enzymatic digestion of glucosides by Rapidase. The substrates and products were analyzed by LC-MS. Enzymatic digestion of sotolone glucoside (**A**) and maple furanone glucoside (**B**) by Rapidase and formation of sotolone (**A**) and maple furanone (**B**), respectively over time.

As furanones show excellent organoleptic properties and controlled release of the aglycones is important when their glucosides are used as slow-release aroma chemicals enzymatic hydrolysis of furanone was analyzed. Since Rapidase is known to rapidly hydrolyze aroma glycosides^51^, this enzyme was used to test the stability of furanone β-D-glucopyranosides (Fig. 6). The glucosides (1 mg/l) were readily degraded by the hydrolase (1 mg/ml) whereby 400 µg of sugar derivatives were completely hydrolyzed by 400 µg of the enzyme within 360 min. Thus, Rapidase efficiently degraded furanone glucosides but probably due to the known instability of the furanones, the theoretical concentrations of the aglycones were not fully recovered.

## Conclusion

Plant glucosyltransferases, because of their pronounced substrate promiscuity are versatile biocatalysts that transfer sugar molecules from activated donor substrates to a range of acceptor molecules. The use of whole-cell UGT biocatalysts is the method of choice to glucosylate small molecules such as aroma chemicals as cofactors are recycled by the cells’ metabolism. This system allows the transformation of a number of low molecular weight compounds at large scale and the production of new-to-nature glycosides, which find promising applications in cosmetics, food, and pharmaceutical industry as profragrances, proflavors, and prodrugs, respectively.

## Methods

### Chemicals

Sotolone (3-hydroxy-4,5-dimethyl-2(5H)-furanone), maple furanone (5-ethyl-3-hydroxy-4-methyl-2(5H)-furanone), furaneol (4-hydroxy-2,5-dimethyl-3(2H)-furanone) and homofuraneol (mixture of 5-ethyl-4-hydroxy-2-methyl-3(2H)-furanone and 2-ethyl-4-hydroxy-5-methyl-3(2H)-furanone) and other reagents were purchased in analytical grade from Sigma-Aldrich, Steinheim (Germany).

### UGT-library

The UGT library consisted of the following 49 UGTs: UGT71A33, UGT71A34a, UGT71A35, UGT71A44, UGT71AJ1, UGT71K3a, UGT71K3b, UGT71W2, UGT72AY1, UGT72B27, UGT730A1, UGT73A15, UGT73AR2, UGT73B23, UGT73C5, UGT73E12, UGT73E13, UGT73E14, UGT73E15, UGT73E16, UGT75L22, UGT75L24, UGT75T1, UGT76Q2, UGT76Q3, UGT76Q4, UGT84A14, UGT84A16, UGT84A41, UGT84A42, UGT84A43, UGT84A44, UGT84A45, UGT84A46, UGT84A49, UGT85A69a, UGT85A69co, UGT85A70, UGT85A72, UGT88A12, UGT88A20, UGT88A21, UGT88A22, UGT88A23, UGT88A24, UGT88A25, UGT88A26, UGT88A27, and UGT92G6 (http://prime.vetmed.wsu.edu/resources/udp-glucuronsyltransferase-homepage). The corresponding genes were cloned into pGEX-4T1 and transferred into *Escherichia coli* W^52^.

### Screening of UGTs

For the initial screening of the enzymatic activity of the UGT-library towards the substrates sotolone, maple furanone, furaneol and homofuraneol in a small scale biotransformation process, a 50 ml overnight culture of *E*. *coli* W:pGEX-4T1:UGT in M9 minimal media containing 1% sucrose with 50 mg/L ampicillin was prepared. After measuring the OD_600_, 25 ml of the bacterial culture was centrifuged at 5,292 × *g*, and RT, for 15 min and the pellet was resuspended in M9 minimal media containing 0.2 mM isopropyl β-D-1-thiogalactopyranoside (IPTG) to OD_600_ 1. Five ml of the bacterial suspension was transferred to a 25 ml deep well microtiter plate (HJ-BIOANALYTIK GmbH, Erkelenz, Germany), and incubated for 3 hr at 18 °C and 300 rpm. The biotransformation was started by adding substrate at a final concentration of 1 g/L. After one day of incubation with shaking at RT, 0.5% sucrose was added to the culture. On the second day, the culture supernatant was centrifuged at 12,045 × *g* for 2 min, diluted 1:10 and analyzed for glucoside formation by LC-UV-MS. For relative quantification, the UV-peak areas at 232 nm for sotolon glucoside and maple furanone glucoside and 280 nm for furaneol glucoside and homofuraneol glucoside were used and the UGT with the highest product formation (peak area) was set to 100% (relative biotransformation rate).

### Enzymatic digestion of glucosides

Four hundred µg purified glucoside (1 mg/ml glucoside solution) was incubated with 400 µg Rapidase AR 2000 (DSM Food Specialties Beverage Ingredients, MA Delft, The Netherlands) in water at RT. Aliquots of 20 µl were taken over the time (0–360 min) and the hydrolysis reaction was stopped by heating the mixture for 10 min at 75 °C. The glucoside and aglycone concentration was determined with standard curves by LC-UV-MS.

### Product identification using LC-UV-MS

The glycosides were identified according to the method described in^53^. The LC System (quaternary pump and variable wavelength detector) were all from Agilent 1100 (Bruker Daltonics, Bremen, Germany). The glycosides were separated by a LUNA C18 100 A 150 × 2 mm (Phenomenex, Aschaffenburg, Germany) with a flow rate of 0.2 ml/min. Sotolone and maple furanone were monitored at 232 nm, furaneol and homofuraneol at 280 nm. The binary gradient system consisted of solvent A, water with 0.1% formic acid and solvent B, 100% methanol with 0.1% formic acid with following gradient program: 0–3 min: 0–50% B; 3–6 min: 50–100% B; 6–14 min: 100% B; 14–14.1 min: 100–0% B; 14.1–25 min: 0% B. The mass spectra was monitored by a Bruker esquire 3000 plus mass spectrometer with an ESI interface. The ionization voltage of the capillary was 4000 V and the end plate was set to −500 V.

### Whole cell biotransformation on large scale

For the large scale production of sotolone and maple furanone glucosides, 1 L M9 minimal media containing 1% sucrose (50 mg/L ampicillin) was inoculated with *E*. *coli* W:pGEX-4T1:UGT84A45 (sotolone)/UGT71K3a (maple furanone) in a 2 L shaking flask and incubated at 37 °C by shaking at 150 rpm overnight. The UGT expression was induced with 0.2 mM IPTG at an OD_600_ of 1.5–2. Additionally, 1% sucrose and 1× M9 salts were given to the culture and incubated at least for 4 hr at 18 °C by shaking. The biotransformation was started by adding 3 g/L sotolone (or maple furanone) to the culture. The flask was incubated for 8 days at RT by shaking at 150 rpm. Aliquots for OD_600_ measurement and LC-MS analysis were taken at several time points and 0.5% sucrose was fed daily. The biotransformation was completed when the OD_600_ reached its maximum and almost all of the aglycon was transformed. At maximum product level the bacterial cells were removed by centrifugation and the resulting supernatant used for product isolation by solid phase extraction.

### Purification of glucosides

After centrifugation of the biotransformation culture for 40 min at 5,292 × *g* (RT), the supernatant was incubated with an appropriate amount of the Purosorb PAD600 (Purolite Ltd, Llantrisant, UK) over night by shaking. The resin was then thoroughly washed with water and the glucosides were eluted with 5 volumes of methanol by vacuum filtration. The methanol was evaporated and the residue was dissolved in water, followed by a liquid- liquid extraction with ethyl acetate (in total 3 times) to remove remaining aglycone. After evaporation, the residue was dissolved in methanol and treated with activated carbon (1 g/ 100 ml) to remove colored impurities. Finally, the glucosides were dried by evaporation and stored for further experiments at 4 °C.

### NMR analysis

Thirty mg purified glucoside or 15 mg aglycone were solved in 200 µl methanol-d4 (Sigma-Aldrich, Steinheim, Germany), centrifuged at maximum speed for 2 min und evaporated. The residue was solved in 400 µl methanol-d_4_ containing 0.03% (v/v) TMS (Sigma-Aldrich, Steinheim, Germany), transferred to a treated NMR tube und filled up to a final volume of 600 µl. NMR spectra were recorded with a Bruker DRX 500 spectrometer (Bruker, Karlsruhe, Germany). The chemical shifts were referred to the solvent signal. The one-dimensional and two-dimensional COSY, HMQC, and HMBC spectra were acquired and processed with standard Bruker software (XWIN-NMR) and MestreNova software (*mestrelab*.*com*).

## Supplementary information

Supplemental data

## Data Availability

The data that support the findings of this study are available from the corresponding author upon reasonable request.
