# Supplementary material for: Novel biotechnological glucosylation of high-impact aroma chemicals, 3(2H)- and 2(5H)-furanones
Source: Sci Rep. 2019 Jul 29;9:10943. doi: 10.1038/s41598-019-47514-9 (PMC6662797; doi:10.1038/s41598-019-47514-9)
Supplement: Supplementary file 1 — Supplemental data [file 41598_2019_47514_MOESM1_ESM.pdf]

Supplemental Data

Novel biotechnological glucosylation of high-impact aroma chemicals, 3(2H)- and 2(5H)-furanones

Isabelle Effenberger, Thomas Hoffmann, Rafal Jonczyk, Wilfried Schwab

Biotechnology of Natural Products, Technische Universität München, Liesel-Beckmann-Str. 1, 85354 Freising, Germany

Table S1. NMR spectra of sotolone,and marple furanone glucoside. The numbering of carbons and hydrogens is shown in the structures

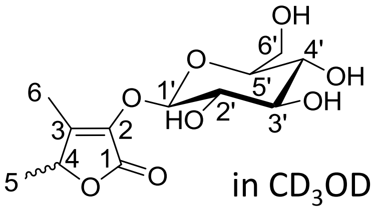

in CD_3OD

atom	δ (ppm)	atom	δ (ppm)
C1	169.14<br>169.23		
C2	144.41<br>144.94		
C3	137.37<br>137.45		
C4	77.56<br>77.68	H4	4.9 q<br>1H
C5	8.69<br>8.77	H5	1.4 dd<br>3H
C6	17.13<br>17.16	H6	2.0 d<br>3H
C1‘	100.81<br>101.08	H1‘	5.1 dd<br>1H
C2‘	73.57<br>73.61	H2‘	3.3–3.4<br>m 1H
C3‘	76.28<br>76.31	H3‘	3.3-3.4<br>m 1H
C4‘	69.76<br>69.79	H4‘	3.3-3.4<br>m 1H
C5‘	76.91<br>76.93	H5‘	3.3-3.4<br>m 1H
C6‘	61.02	H6‘	3.7, 3.8<br>2H

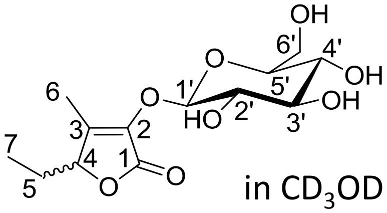

in CD_3OD

atom	δ (ppm)	atom	δ (ppm)
C1	170.79		
C2	144.71<br>145.03		
C3	139.30<br>139.40		
C4	83.04<br>83.16	H4	4.80 m<br>1H
C5	25.79<br>25.85	H5	1.55 2.06<br>m m 2H
C6	10.20<br>10.27	H6	2.01 d 3H
C7	8.05	H7	0.91 dt<br>3H
C1‘	102.52<br>102.22	H1‘	5.1 dd 1H
C2‘	74.97	H2‘	3.3–3.4<br>m 1H
C3‘	77.69	H3‘	3.3-3.4 m<br>1H
C4‘	71.17	H4‘	3.3-3.4 m<br>1H
C5‘	78.26<br>78.33	H5‘	3.3-3.4 m<br>1H
C6‘	62.40	H6‘	3.7, 3.8<br>2H

Table S2. NMR spectra of furaneol glucoside. The numbering of carbons and hydrogens is shown in the structure.

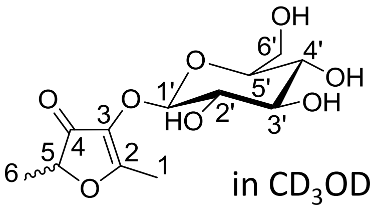

in CD_3OD

atom	δ (ppm)	atom	δ (ppm)
C1	12.83<br>12.86	H1	2.32 d<br>3H
C2	183.10<br>183.34		
C3	133.15<br>133.21		
C4	198.91<br>199.05		
C5	80.99<br>81.02	H5	4.63 m<br>1H
C6	15.12<br>15.19	H6	1.43 d<br>3H
C1‘	103.05<br>103.33	H1‘	4.75 dd<br>1H
C2‘	73.33<br>73.43	H2‘	3.3–3.4<br>m 1H
C3‘	76.27<br>76.31	H3‘	3.3-3.4<br>m 1H
C4‘	69.76<br>69.81	H4‘	3.3-3.4<br>m 1H
C5‘	76.90<br>76.93	H5‘	3.3-3.4<br>m 1H
C6‘	61.04<br>61.09	H6‘	3.7, 3.8<br>dd dd 2H

Table S3. NMR spectra of homofuraneol glucoside isomers derived from 2-ethyl-4-hydroxy-5-methyl-3(2H)-furanone (2-EHMF) and 5-ethyl-4-hydroxy-2-methyl-3(2H)-furanone (5-EHMF). The numbering of carbons and hydrogens is shown in the structures but differs from the IUPAC nomenclature.

2-EHMF

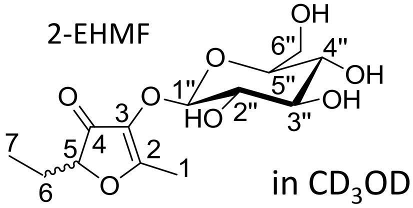

in CD_3OD

5-EHMF

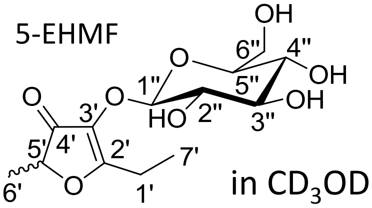

in CD_3OD

atom	δ (ppm)	atom	δ (ppm)
C1	13.91<br>14.12	H1	2.31 d<br>3H
C2	185.08<br>185.22		
C3	135.42<br>135.47		
C4	199.63<br>199.67		
C5	86.43<br>86.66	H5	4.52 m<br>1H
C6	25.21<br>25.36	H6	2.31 2.29<br>d m 2H
C7	8.47<br>8.50	H7	0.96 t 3H
C1‘‘	104.57<br>105.63	H1‘	4.72 t 1H
C2‘‘	73.33<br>73.43	H2‘	3.3–3.4<br>m 1H
C3‘‘	76.90<br>76.92	H3‘	3.3-3.4<br>m 1H
C4‘‘	69.67<br>69.80	H4‘	3.3-3.4<br>m 1H
C5‘‘	76.30	H5‘	3.3-3.4<br>m 1H
C6‘‘	61.01<br>61.09	H6‘	3.66, 3.8<br>m m 2H

atom	δ (ppm)	atom	δ (ppm)
C1‘	22.04	H1‘	2.71 m<br>2H
C2‘	188.17<br>188.30		
C3‘	133.50<br>133.55		
C4‘	200.61<br>200.76		
C5‘	86.21<br>86.43	H5	4.52 m<br>1H
C6‘	16.47<br>16.57	H6‘	1.41 d<br>3H
C7‘	10.29	H7	1.22 t 3H
C1‘‘	104.30<br>104.53	H1‘	4.74 t 1H
C2‘‘	73.35<br>73.47	H2‘	3.3–3.4<br>m 1H
C3‘‘	76.90<br>76.92	H3‘	3.3-3.4<br>m 1H
C4‘‘	69.78<br>69.83	H4‘	3.3-3.4<br>m 1H
C5‘‘	76.34	H5‘	3.3-3.4<br>m 1H
C6‘‘	61.05	H6‘	3.66, 3.8<br>m m 2H

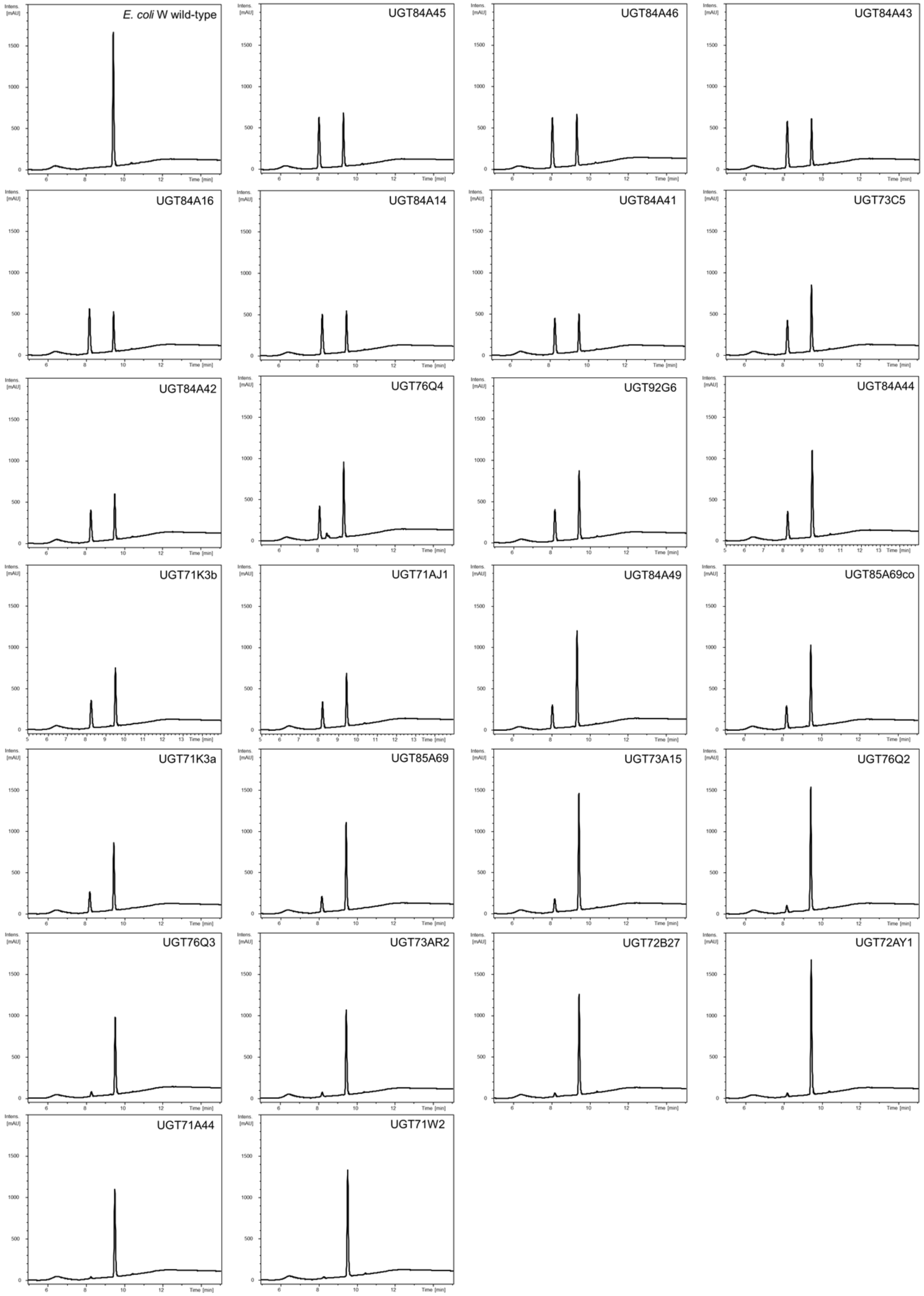

Figure S1. Positive screening results of UGTs expressed *in E. coli* W with sotolone.
